# How Anacetrapib Inhibits the Activity of the Cholesteryl Ester Transfer Protein? Perspective through Atomistic Simulations

**DOI:** 10.1371/journal.pcbi.1003987

**Published:** 2014-11-20

**Authors:** Tarja Äijänen, Artturi Koivuniemi, Matti Javanainen, Sami Rissanen, Tomasz Rog, Ilpo Vattulainen

**Affiliations:** 1 Department of Physics, Tampere University of Technology, Tampere, Finland; 2 VTT Technical Research Center of Finland, Espoo, Finland; 3 MEMPHYS – Centre for Biomembrane Physics, University of Southern Denmark, Odense, Denmark; University of Illinois, United States of America

## Abstract

Cholesteryl ester transfer protein (CETP) mediates the reciprocal transfer of neutral lipids (cholesteryl esters, triglycerides) and phospholipids between different lipoprotein fractions in human blood plasma. A novel molecular agent known as anacetrapib has been shown to inhibit CETP activity and thereby raise high density lipoprotein (HDL)-cholesterol and decrease low density lipoprotein (LDL)-cholesterol, thus rendering CETP inhibition an attractive target to prevent and treat the development of various cardiovascular diseases. Our objective in this work is to use atomistic molecular dynamics simulations to shed light on the inhibitory mechanism of anacetrapib and unlock the interactions between the drug and CETP. The results show an evident affinity of anacetrapib towards the concave surface of CETP, and especially towards the region of the N-terminal tunnel opening. The primary binding site of anacetrapib turns out to reside in the tunnel inside CETP, near the residues surrounding the N-terminal opening. Free energy calculations show that when anacetrapib resides in this area, it hinders the ability of cholesteryl ester to diffuse out from CETP. The simulations further bring out the ability of anacetrapib to regulate the structure-function relationships of phospholipids and helix X, the latter representing the structural region of CETP important to the process of neutral lipid exchange with lipoproteins. Altogether, the simulations propose CETP inhibition to be realized when anacetrapib is transferred into the lipid binding pocket. The novel insight gained in this study has potential use in the development of new molecular agents capable of preventing the progression of cardiovascular diseases.

## Author Summary

Cardiovascular disease is a leading cause of morbidity and mortality in Western societies. One of the most encouraging treatment methods to prevent the generation and progression of cardiovascular disease is the elevation of high density lipoprotein (HDL) levels in circulation, as high HDL levels have been found to correlate negatively with the risk of cardiovascular disease. HDL elevation is attainable through inhibition of cholesteryl ester transfer protein (CETP). A novel molecular agent, anacetrapib, fulfills the requirements with an acceptable side-effect profile. In this study, our objective is to gain more detailed information regarding the interactions between CETP and anacetrapib in order to unlock the inhibitory mechanism of the drug that has, to date, remained unclear. Our results point out the primary binding site of anacetrapib and highlight the ability of the drug to regulate the structure-function relationship of those structural regions of CETP that are considered important in CETP inhibition. Our findings could be exploited in the development of new and more efficient molecular agents against cardiovascular disease.

## Introduction

Cholesteryl ester transfer protein (CETP) is a 476-residue-long hydrophobic glycoprotein that transports cholesteryl esters (CEs), triglycerides, and phospholipids between high density lipoprotein (HDL) and other lipoprotein fractions in human blood plasma [1]. To be more specific, CETP exchanges CEs of HDL particles to triglycerides of very low density lipoproteins (VLDL) and low density lipoproteins (LDL), thus increasing the amount of triglycerides in HDL, leading to more rapid catabolism of HDL particles.

**Figure 1:**
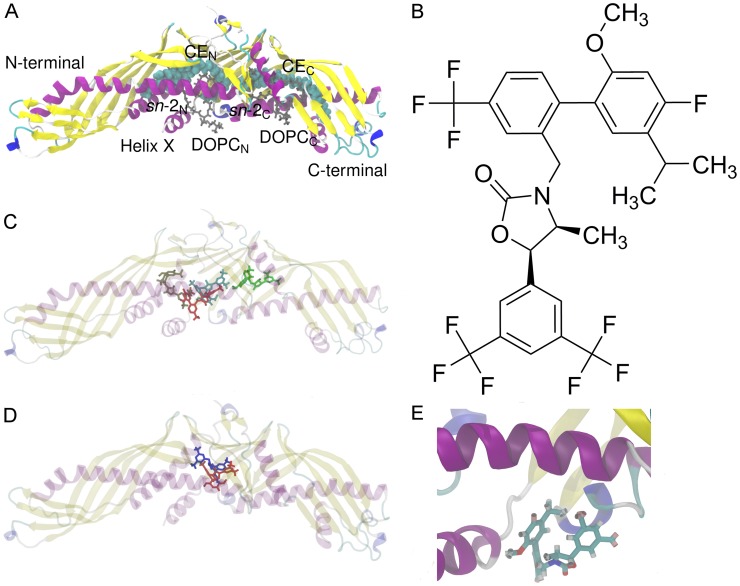
Structures of CETP and anacetrapib and results from molecular docking. A) X-ray structure of human CETP. The two DOPCs (gray) plug the tunnel openings that lead to the hydrophobic tunnel where two CEs (cyan spheres) are located. The *sn*-chains of DOPCs, the N- and C-terminals, as well as helix X are labeled. B) The atomic formula of anacetrapib. Anacetrapib is a 1–3-oxazolidin-2-one based CETP inhibitor. C) The most probable binding sites and conformations of anacetrapib within the structure of CETP obtained from molecular docking calculations. The binding energies for the binding sites of red, brown, cyan, and green ligands are −47.7 kJ mol^−1^, −46.4 kJ mol^−1^, −48.5 kJ mol^−1^, and −46.9 kJ mol^−1^, respectively. D) The most probable binding site of anacetrapib gained from docking calculations matched with the recently published X-ray structure of CETP with bound torcetrapib. Anacetrapib is presented with red and torcetrapib with blue color. E) Simulation snapshot from simulation S2-1nm. While moving at the N-terminal tunnel opening, anacetrapib aligns itself to a tighter conformation through the orientation of trifluoromethyl- and methyl groups close to each other.

CETP (Figure 1) carries CEs within a 6-nm-long hydrophobic tunnel that traverses the core of the molecule [2]. The tunnel has two distinct openings, and in the crystal structure [2] both of them are plugged by a dioleoylphosphatidylcholine (DOPC) molecule (Figure 1A). The lipid exchange mechanism of CETP is poorly understood. One plausible mechanism is the so-called shuttle mechanism [1], [3], in which CETP binds only one lipoprotein at a time. CETP attaches to the surface of a lipoprotein via its concave surface where also the two tunnel openings reside [2], [4]. The openings are expected to serve as passages to the flow of neutral lipids (CEs and triglycerides) between the particles, and their location supports the view that the concave surface is the only site able to bind lipoproteins, since other surfaces of the protein lack direct access to the tunnel. Further, the inherent curvature of CETP matches well with the curvature of HDL particles that may result from the fact that a major part of CETP has been shown to be associated with HDL due to higher binding affinity compared with plasma LDL or VLDL [1]. However, the molecular details driving the diffusion of lipids into and out from CETP require further elucidation. Previous experimental studies indicate that helix X located at the C-terminal domain of CETP is detrimental for the neutral lipid exchange, but not for the exchange of phospholipids [5], [6]. Helix X has been proposed to act as a lid conducting the exchange of lipids by alternating its open and closed states [2], [4]. In a recent molecular dynamics simulation study it was shown that after the attachment of CETP to lipoprotein surface, helix X is able to fold into the hydrophobic tunnel and interact with the CETP-bound CE [4]. After the lipids have been exchanged, the tunnel openings are plugged by phospholipids followed by the detachment of CETP from the lipoprotein surface. Meanwhile, in addition to the shuttle mechanism, another transportation mechanism has also been suggested. Here, CETP forms a ternary complex with two lipoprotein particles, and lipids somehow diffuse from one lipoprotein to another through the hydrophobic tunnel [7].

The interest towards CETP and its lipid transfer functions came to the forefront after notable associations between decreased CETP activity, decreased LDL-cholesterol level, increased HDL-cholesterol level, and resistance to atherosclerosis [8]. Atherosclerosis is a leading cause of morbidity and mortality in Western societies, and several clinical trials have shown that low HDL levels correlate with the risk of atherosclerosis due to antiatherogenic properties of HDL to remove cholesterol from atherosclerotic plaques back into liver to be recycled or secreted into bile [9], [10]. Pharmacological CETP inhibition has therefore emerged as a prime target to modulate HDL levels, with an objective to become a potential treatment strategy for preventing various cardiovascular diseases. Unfortunately, the road of CETP inhibition has had a turbulent beginning due to the failure of its two first compounds, torcetrapib and dalcetrapib. Torcetrapib increased the level of HDL as was hoped, but additionally it also increased the blood pressure as well as the mortality rate [11]. Due to these reasons, all clinical trials concerning torcetrapib had to be terminated. In the case of dalcetrapib, the trials were halted for futility as only modest increases in HDL levels were reached [12]. However, subsequent studies indicate that CETP remains a valid target since the lethal side effects seen with torcetrapib are unrelated with CETP inhibition and may not be shared by the other members of CETP inhibitors. A real breakthrough was reached at the end of 2010 when a new variant of the drug, anacetrapib (Figure 1B), was found to inhibit CETP with an acceptable side-effect profile [13]. In addition to anacetrapib, a competing inhibitor currently under phase III clinical development, evacetrapib, has already been shown to dose-dependently inhibit CETP and to increase HDL without severe cardiovascular events [14].

Despite the promising start achieved in the anacetrapib-based CETP inhibition, the precise inhibitory mechanism behind the agent is yet to be proven, regardless of the extensive research efforts its clarification has demanded. Classically, drugs inhibit or promote the functions of an enzyme or a receptor by binding e.g. to the active site thus blocking the binding of a ligand. However, in the case of CETP inhibitors, it has been demonstrated that they promote the formation of CETP-HDL complex, indicating that the current inhibitors do not only compete with CEs and triglycerides in CETP-binding but, additionally, they also hamper the detachment of CETP from HDL surface [15], [16]. The reported ability of anacetrapib, torcetrapib and dalcetrapib to increase the binding affinity between CETP and HDL, and hereby to promote the inhibition of lipid exchange between the particles, does not clarify the actual mechanism of action nor the possible structural changes required. However, explanations concerning especially the role of phospholipids and helix X in this regard have been proposed but the details at an atomistic level are largely unknown, and thus the understanding of the molecular basis of inhibition is quite limited. Recently, the X-ray structures of CETP were published with bound torcetrapib and one of its analogs [17]. Surprisingly, the structure showed that torcetrapib is able to bind CETP even if both CEs are bound to CETP. However, the C-terminal phospholipid was not present in the structure suggesting that torcetrapib abolishes the binding of a phospholipid to the C-terminal tunnel opening. This, again, could make the detachment of CETP from the surface of a lipoprotein more unfavorable, thus stabilizing the CETP-HDL complex. It remains to be seen if this holds for anacetrapib, since it has a similar chemical structure compared with torcetrapib [18]. Another mechanism that has been suggested to stabilize the CETP-lipoprotein complex is the ability of anacetrapib to act as glue between the particles [16].

The above findings and suggestions are insightful and encouraging, but call for better understanding of the inhibitory mechanism of anacetrapib, as well as of the lipid transfer functions related with CETP inhibition. In this regard, our objective is to perform atomistic molecular dynamics simulations to obtain detailed information linking the two processes. Here we have studied the interactions between anacetrapib and CETP with different lipid compositions to gather novel information regarding the inhibition of CETP. Previous studies of HDL-like lipid droplets [4], [19], HDL [20], and LDL [21] have shown that atomistic and coarse-grained simulations of lipoproteins and related transfer proteins can provide significant insight into their nanoscale properties and the mechanisms associated with lipid transfer. Understanding the lipid transfer functions of the protein, as well as the mechanisms behind CETP inhibitors, are important to realize in order to develop safe and efficacious treatment methods for the pharmacological raising of HDL cholesterol levels.

We find that anacetrapib has a strong affinity for the region of the N-terminal tunnel opening. The primary binding site of anacetrapib turns out to reside in the tunnel inside CETP, near the residues surrounding the N-terminal opening. Free energy calculations show that when anacetrapib resides in this area, it hinders the ability of CE to diffuse out from CETP. The simulations further bring out the ability of anacetrapib to regulate the structure-function relationships of phospholipids and helix X, the latter representing the structural region of CETP important to the process of neutral lipid exchange with lipoproteins. Altogether, the simulations propose CETP inhibition to be realized when anacetrapib is transferred into the lipid binding pocket. The present study serves as a solid foundation for future studies concerning interactions between anacetrapib and CETP-lipoprotein complexes.

## Results

### Molecular docking reveals that the most stable binding sites of anacetrapib are inside the tunnel of CETP

To find the most probable sites from the crystal structure of CETP where anacetrapib would favor to attach, we used the energy-minimized and flexible structure of anacetrapib together with the crystal structure of CETP. Here, the aim was not to identify the possible binding poses of anacetrapib, but rather to show the most probable binding sites of the drug molecule. The results are illustrated in Figure 1C and show that, according to the docking calculations, the most favorable binding site for anacetrapib resides in the hydrophobic tunnel of the protein. For ligands colored with red, brown, cyan, and green, the respective binding energies are −47.7 kJ mol^−1^, −46.4 kJ mol^−1^, −48.5 kJ mol^−1^, and −46.9 kJ mol^−1^. In order to further validate the binding site of anacetrapib, the recently published X-ray structure of CETP with bound torcetrapib [17] was matched with the most probable binding site of anacetrapib gained from the docking calculations, see Figure 1D. The binding sites are in good accordance with each other, although CEs were absent from our calculations. In conclusion, docking calculations suggest that anacetrapib could either compete with CETP-bound CEs or phospholipids in binding, or lock them to reside more tightly inside CETP. Below we discuss this topic in more detail based on the free energy calculations that we have carried out to unlock this issue.

### Self-assembly simulations indicate that van der Waals interactions drive the formation of CETP-anacetrapib complexes for intramolecular distances less than 3 nm

**Figure 2:**
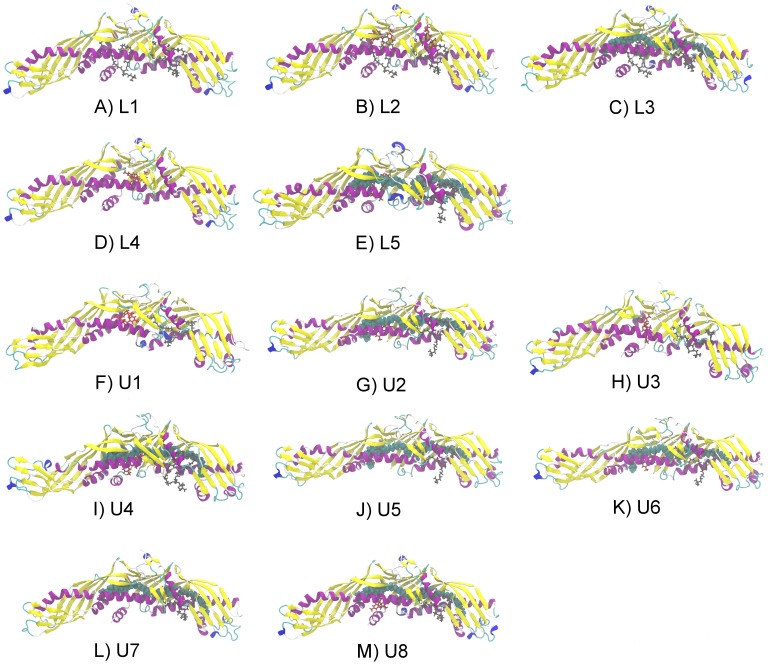
Initial configurations of the simulated systems. Starting configurations of the A)–E) longer 200 ns atomistic simulations as well as the F)–M) free energy calculations where DOPCs are presented as gray stick models, CEs as cyan spheres and anacetrapibs as red stick models. For clarity, the starting configurations of the 20 ns atomistic simulations are not presented.

We carried out ten 20 ns atomistic simulations for fully hydrated systems containing CETP with anacetrapib placed outside but in a close proximity with the protein (S1-helix, S2-1nm, S3-2nm, S4-3nm, S5-4nm, S6-convex, S7-1N, S8-2N, S9-1C, S10-3C; see Table 1 and Materials and Methods) to study the self-assembly process as well as the interactions between anacetrapib and the concave surface of CETP. The initial configurations were constructed to reflect random initial conditions to better correspond to the biological environment of the particles. We also performed five 200 ns simulations involving CETP with different interior lipid and anacetrapib compositions (L1, L2, L3, L4, L5; see Table 1 and Figure 2) to investigate the effects of anacetrapib to the conformational properties of CETP and bound phospholipids. The simulations without anacetrapib (L1, L3) served as control simulations to enable a more elaborate specification of the structural changes induced by the drug. In addition to the molecular dynamics simulations, we conducted eight umbrella sampling (free energy) simulations where both anacetrapib (U1, U2, U3, U4; see Table 1 and Figure 2) and the N-terminal CE (U5, U6, U7, U8; see Table 1 and Figure 2) were pulled out from the hydrophobic tunnel of CETP through the N-terminal opening. The purpose was to determine whether the anacetrapib located inside the tunnel has an influence on the diffusion of CE out from CETP, and to see the effect of helix X in this process.

**Table 1:** Performed simulations and their setups.

Simulation	System setup	Time (ns)
S1-helix	Anacetrapib 1 nm from helix X	20
S2-1nm	Anacetrapib 1 nm from tunnel openings	20
S3-2nm	Anacetrapib 2 nm from tunnel openings	20
S4-3nm	Anacetrapib 3 nm from tunnel openings	20
S5-4nm	Anacetrapib 4 nm from tunnel openings	20
S6-convex	Anacetrapib 3 nm from the convex back of CETP	20
S7-1N	Anacetrapib 1 nm from the N-terminal end	20
S8-2N	Anacetrapib 2 nm from the N-terminal end	20
S9-1C	Anacetrapib 1 nm from the C-terminal end	20
S10-3C	Anacetrapib 3 nm from the C-terminal end	20
L1	2 DOPCs	200
L2	2 DOPCs, 2 anacetrapibs inside the tunnel	200
L3	2 DOPCs, 2 CEs inside the tunnel	200
L4	Anacetrapib inside the tunnel	200
L5	DOPC_C_, 2 CEs and one anacetrapib inside the tunnel	200
U1	DOPC_C_, helix X removed, anacetrapib inside the tunnel	160
U2	DOPC_C_, helix X removed, 2 CEs and one anacetrapib inside the tunnel	160
U3	DOPC_C_, anacetrapib inside the tunnel	160
U4	DOPC_C_, 2 CEs and one anacetrapib inside the tunnel	160
U5	DOPC_C_, helix X removed, 2 CEs inside the tunnel	160
U6	DOPC_C_, helix X removed, 2 CEs and one anacetrapib inside the tunnel	160
U7	DOPC_C_, 2 CEs inside the tunnel	160
U8	DOPC_C_, 2 CEs and one anacetrapib inside the tunnel	160

All simulations include CETP.

**Figure 3:**
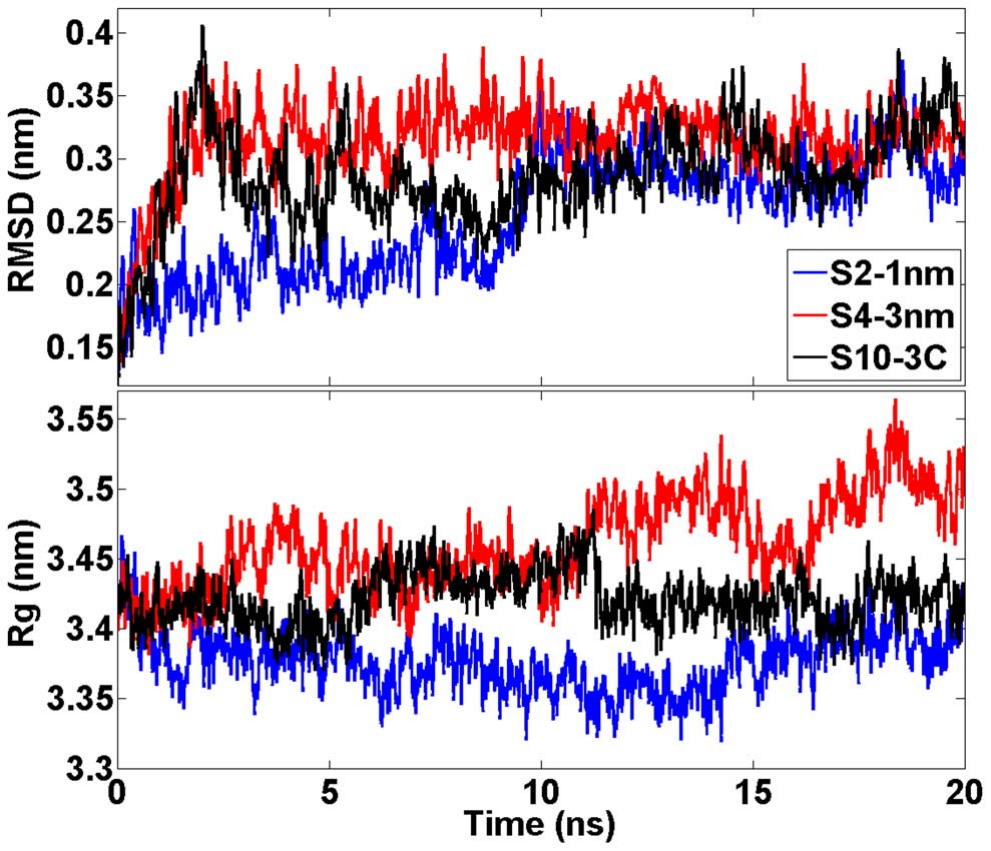
Structural measures of CETP during short simulations. RMSD and radii of gyration profiles for CETP during the short 20 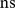 simulations for three of the studied systems. Results for the other systems are largely similar.

**Figure 4:**
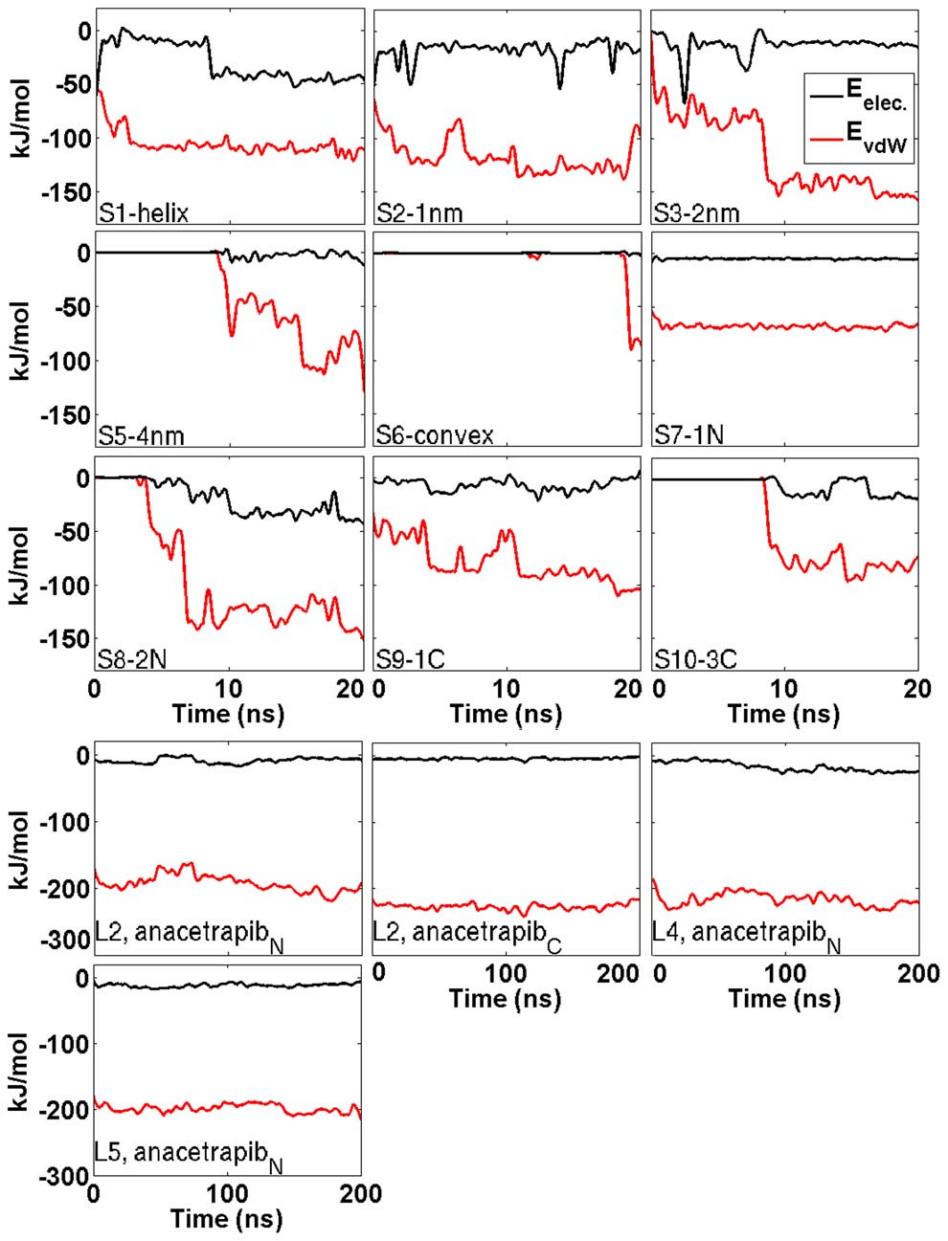
Interactions between CETP and anacetrapib. The van der Waals and electrostatic interaction energies between CETP and anacetrapib in the simulations performed for 20 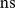 (upper three rows). Additionally also the interaction energies concerning simulations L2, L4, and L5 are presented (bottom two rows). Interaction energies for the simulation S4-3nm are not presented due to small values. Interaction energies between the molecules are observable when anacetrapib enters the 1 nm cutoff region during the course of simulations.

Root mean square deviation (RMSD) profiles of 20 ns simulations indicate that the structures do not deviate considerably from the corresponding X-ray structure (Figure 3). The radii of gyration fluctuated between 3.33 and 3.54 nm in each simulation (Figure 3). The spatial density maps illustrated in Figure S1 (see Supporting Information (SI)) reveal a disordered motion of the drug molecule around CETP. The distance between the particles ranged from 1 to 2 nm in the simulations S1-helix, S2-1nm, S3-2nm, S7-1N, S8-2N, and S9-1C, and from 3 to 4 nm in the simulations S4-3nm, S5-4nm, S6-convex, and S10-3C. A change in the distance from 2 to 3 nm weakens considerably the interactions between the CETP and anacetrapib and, as a consequence, anacetrapib experiences random movement due to thermal fluctuations. These findings are supported by the interaction energies calculated between the particles (Table 2, Figure 4). Table 2 presents the interaction energies averaged over the course of the simulations, whereas Figure 4 depicts the energies as a function of simulation time. It is apparent that the main force to drive the binding of anacetrapib to CETP in S1-helix, S2-1nm, S3-2nm, S7-1N, S8-2N, and S9-1C is the weak van der Waals interactions. In the remaining simulations both the van der Waals electrostatic interaction forces are substantially weaker.

**Table 2:** Average interaction energies and their standard deviations between CETP and anacetrapib.

Simulation	E_vdw_ (kJ/mol)	E_electrostatic_ (kJ/mol)
S1-helix	−105.7±3.8	−30.0±7.8
S2-1nm	−117.8±4.4	−18.4±2.0
S3-2nm	−115.8±17.0	−13.7±1.4
S4-3nm	Small figure	Small figure
S5-4nm	−38.4±19.0	−1.7±0.7
S6-convex	−4.5±4.3	−0.1±0.1
S7-1N	−68.1±0.4	−5.0±0.1
S8-2N	−93.1±25.0	−20.1±6.8
S9-1C	−79.1±8.2	−7.4±2.0
S10-3C	−45.1±19.0	−5.9±2.9
L2, N-terminal anacetrapib	−193.0±5.5	−8.7±1.7
L2, C-terminal anacetrapib	−228.0±1.1	−4.7±0.3
L4	−217.1±3.2	−17.1±3.3
L5	−199.4±1.4	−12.1±0.9

It is worth to notice that in S1-helix, S2-1nm, S3-2nm, S7-1N, S8-2N, and S9-1C, the movement of anacetrapib is highly similar, since the drug moves at the N-terminal tunnel opening mainly around residues Arg197, Ser431, Lysh432, Gly433, Ser435, and Hisb462, which is in good agreement with our results based on molecular docking, concerning especially the binding site of the red ligand (Figure 1C, Figure S1).

The visualization of the simulation trajectories also revealed that while the drug moved at the N-terminal opening, the trifluoromethyl- and methyl groups of the drug oriented close to each other (Figure 1E). This indicates that the drug aligned itself to a tighter conformation suggesting a possible movement into the tunnel. The observed finding is supported by the interaction energies calculated from the simulations where anacetrapib was transferred into the hydrophobic tunnel of CETP (L2, L4, L5; Table 2, Figure 4). The strength of van der Waals interactions between the particles is two to two-and-a-half times stronger inside the tunnel than at the N-terminal opening, or at the N- and C-terminal ends of the protein. This strongly suggests that CETP inhibition is enabled when the drug enters the protein, and the drug interacts with the structural regions of CETP important to lipid exchange from the hydrophobic cavity. The proposed movement is further supported by the lack of a significant number of hydrogen bonds between the protein and the drug. Anacetrapib was noticed to form separate hydrogen bonds with CETP while moving at the N-terminal tunnel opening, implicating that when a new bond was formed, the previous one was broken. This implies a weak attachment to CETP, and thus an unobstructed movement of the drug.

The present findings based on a self-assembly process highlight the importance of thermal diffusion together with electrostatic and van der Waals interactions during the formation of CETP-inhibitor complexes. Thermal motion predominates for distances above 3 nm between anacetrapib and the tunnel opening of CETP, and only for shorter scales the direct interactions between the molecules become strong enough to drive the complex formation process. Furthermore, the affinity of anacetrapib towards the concave surface of CETP is evident when the distance between the particles is taken into consideration. On the basis of the above findings, we propose that the primary binding site of anacetrapib resides inside the hydrophobic tunnel of the protein, near the residues surrounding the N-terminal tunnel opening, including helix X.

### Free energy profiles indicate a reduced ability of cholesteryl ester to diffuse out from CETP as anacetrapib locates inside the tunnel

**Figure 5:**
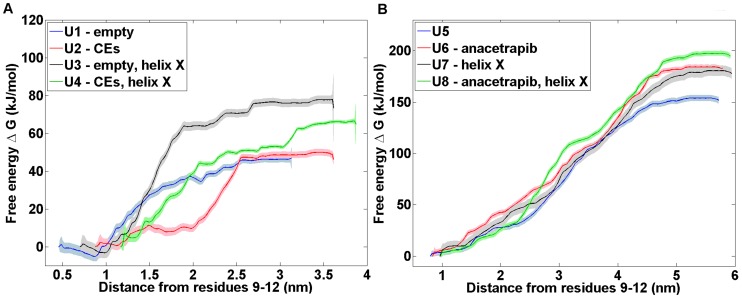
Free energy profiles of anacetrapib and cholesteryl ester. Free energy profiles of the umbrella sampling simulations. Both A) anacetrapib and B) cholesteryl ester were pulled out from the structure of CETP through the N-terminal tunnel opening to the water phase. CETP residues Cysh9, Arg10, Ile11, and Thr12 were used as the reference pull group due to their parallel alignment with the N-terminal tunnel opening.

Computation of free energy profiles is a major computational challenge in general. The case considered here is not an exception. To get the free energy profiles shown in Figure 5 through umbrella sampling simulations required substantial computer resources (see Materials and Methods). Despite this, the obtained profiles are not fully converged. This issue typically arises from inadequate sampling of regions of conformational space that are likely separated by a large barrier. While the data presented here represents largely the state of the art, we yet wish to stress that the best we can extract from the data are suggestive trends.

The obtained free energy profiles (Figure 5) indicate strong attachment between anacetrapib and CETP. They further point towards the possibly hindered ability of the CE molecule to diffuse from CETP to the water phase when both the drug molecule and two CE lipids reside in the hydrophobic tunnel (Figure 5). The corresponding free energy barriers range up to 65 kJ mol^−1^ for anacetrapib, and between 184 and 197 kJ mol^−1^ for CE (when anacetrapib blocks the path).

The free energy barriers found here, as CE is pulled out from CETP, are profoundly high. This results from the transition path that takes CE completely into the water phase. Importantly, Figure 5 also suggests that the increase in free energy is rather modest at short distances when CE is still inside the hydrophobic environment of CETP. Consequently, the free energy barriers are expected to become lower when CETP binds to a lipoprotein surface, hence facilitating the diffusion and exchange of lipids between the particles.

It is worth to notice that the presence of helix X seems to have an influence on both the binding strength of anacetrapib and the diffusion ability of CE, since both molecules are pulled out from the hydrophobic tunnel more easily when helix X is removed from the structure of CETP. Additionally, when also the drug molecule is removed, the free energy barrier of CE movement towards the water phase appears to be the lowest, 153 kJ mol^−1^. The results point towards the important role of helix X in assisting the lipid exchange, and highlight the possible inhibitory mechanism of anacetrapib as the movement of CE outside from CETP could be hindered in the presence of the drug molecule.

There are two thermodynamic cycles for binding and unbinding both anacetrapib and CE present in the free energy calculations, namely, the one with the absence of helix X from the structure of CETP (U1/U6/U2/U5) and the other with the presence of helix X in the structure of the protein (U3/U8/U4/U7). With the current data, these cycles result in values of −28±20 kJ mol^−1^ for the first and −30±20 kJ mol^−1^ for the latter cycle, where one is tempted to expect for a value of zero. However, the binding site of anacetrapib is not identical in systems where the hydrophobic tunnel is empty (U1, U2) or contains two CEs (U5, U6). To be more specific, the binding site of anacetrapib resides deeper in the hydrophobic tunnel of CETP as the tunnel does not include lipids, while in the presence of these lipids the binding site of the drug molecule resides closer to the N-terminal tunnel opening. Hence, with the present systems, it is not possible to obtain a thermodynamic cycle that would result in a value of zero, and the values of the thermodynamic cycles given above therefore partly stem from this fact, and partly from inadequate sampling. Given the considerable computing resource to generate the data in Figure 5 (see Materials and Methods), we consider it quite unfeasible to reduce the systematic error, but this is not an issue since the main conclusions that can be drawn from Figure 5 are evident based on the data shown.

### CETP-bound anacetrapib induces conformational fluctuations to phospholipids

**Figure 6:**
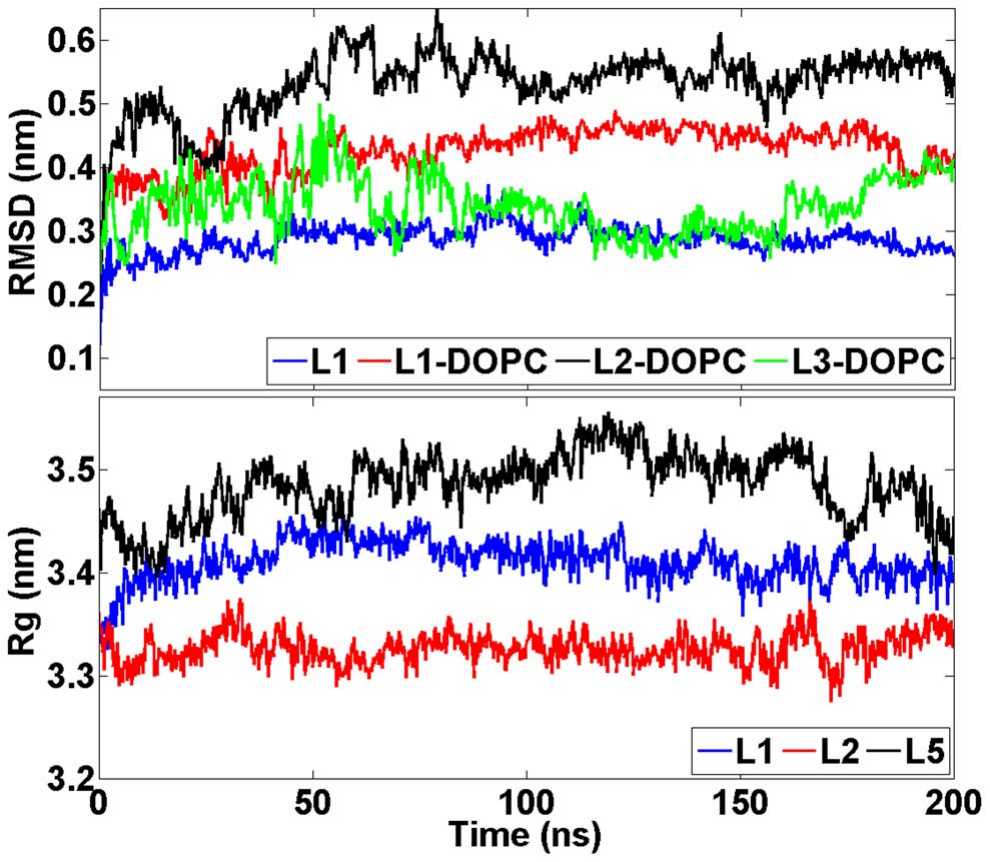
Structural measures of CETP during long simulations. RMSD and radii of gyration profiles for CETP during the long 200 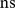 simulations, the systems L1–L3 and L5 shown here being suggestive of the data. The RMSD graph illustrates separately the RMSD values for the CETP bound DOPCs.

**Figure 7:**
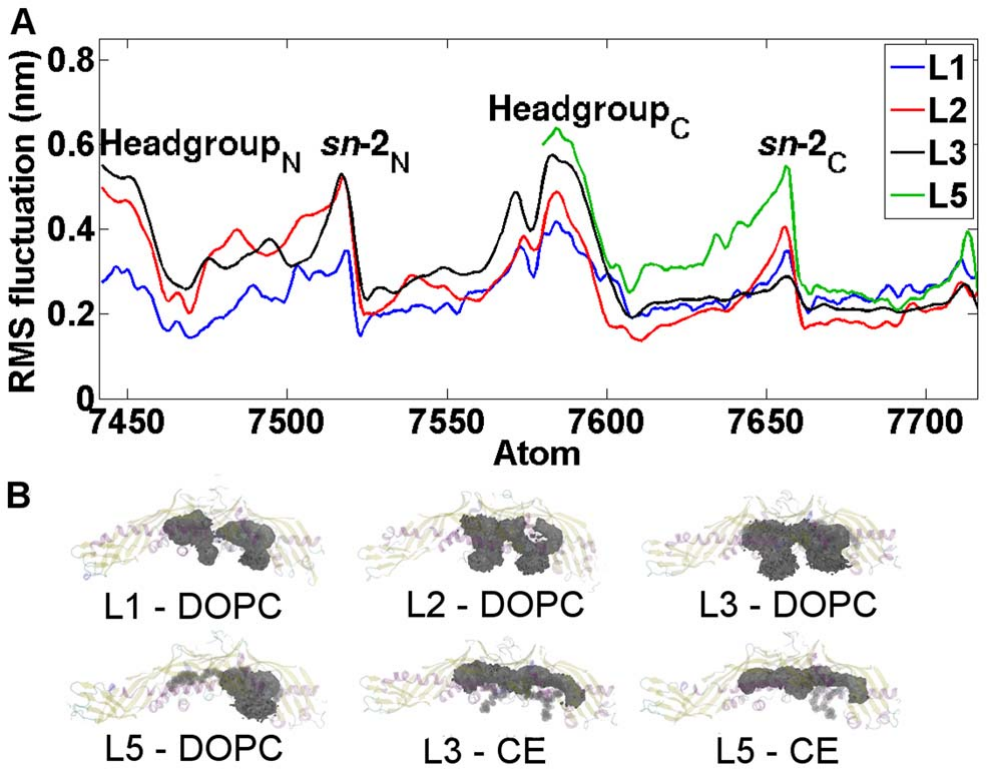
Dynamical properties of CETP bound lipids. A) Atomic RMS fluctuation profiles for DOPCs (atoms 7441–7716) during the long 200 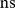 simulations. The peaks indicate the atoms that fluctuate the most over the course of simulations. The positions of DOPC headgroups and *sn*-2 chains with respect to atom numbers are labeled. The headgroup _N_ and the *sn*-2_N_ chain correspond with the headgroup and the *sn*-2 chain of the N-terminal DOPC, while the headgroup _C_ and the *sn*-2_C_ chain correspond with the C-terminal DOPC, see Figure 1A. B) Spatial density maps for DOPCs and CEs. The map reveals the movement of the corresponding lipid inside the binding pocket of CETP.

RMSD profiles of atomistic 200 ns simulations indicate that the protein structures do not deviate considerably from the corresponding X-ray structures (Figure 6). The RMSD values of DOPCs are also plotted in Figure 6, and the profiles depict increased conformational alterations of DOPCs when the drug molecule is transferred into the hydrophobic tunnel of CETP: the RMSD fluctuates between 0.27 and 0.47 nm without the drug (L1, L3), and between 0.26 and 0.61 nm with the drug (L2). This finding is further supported by the atomic RMS fluctuations and spatial density maps calculated for DOPCs (Figure 7). Maps confirm that DOPCs, especially their head groups and *sn*-2 chains, experience considerable wobbling in the presence of anacetrapib (L2) compared with the absence of the drug (L1). However, there is a contradiction when comparing the RMSD and RMSF curves of DOPC in simulations L1 and L3. The RMSD appears more stable in L3 (Figure 6), but the RMSF is more stable in L1 (Figure 7). A reason for this could be that DOPC shifts from its initial position in L1 and is then stabilized there. Nonetheless, the results are in good accordance with the interaction energies calculated between CETP and the associated lipids (DOPCs and CEs), as well as between the lipids and anacetrapib (Table 3). As Table 3 illustrates, the strength of interactions between CETP and DOPCs are the strongest in the absence of anacetrapib (L1). Furthermore, as the interactions between DOPCs and CEs (L3) seem to be stronger than between the lipids and anacetrapib (L2, L5), it is evident that anacetrapib induces high fluctuations to phospholipids. This provides compelling evidence that the drug interacts with phospholipids, and, as a consequence, could hinder the binding of DOPCs to the tunnel openings, which could play a role in the stabilization of the CETP-lipoprotein complex.

**Table 3:** Average interaction energies and their standard deviations between different particles in longer 200 ns simulations.

Simulation	Molecule-molecule pair	E_vdw_ (kJ/mol)	E_electrostatic_ (kJ/mol)
L1	CETP-DOPC	−752.8±12.0	−229.8±6.9
L2	CETP-DOPC	−613.3±5.5	−263.5±12.0
L2	DOPC-anacetrapib	−70.7±2.1	−2.8±1.0
L3	CETP-DOPC	−590.0±5.4	−215.0±24.0
L3	CETP-CE	−666.4±3.2	4.6±0.4
L3	DOPC-CE	−95.0±1.1	2.3±0.1
L5	CETP-DOPC	−299.4±8.2	−80.0±8.6
L5	CETP-CE	−604.7±4.8	6.5±0.4
L5	DOPC-CE	−40.7±3.4	1.0±0.2
L5	DOPC-anacetrapib	Small figure	Small figure
L5	CE-anacetrapib	−70.5	−7.5

As the interaction energies between DOPC and CE molecules imply (Table 3), the fluctuations of the same order of magnitude were observed also when two CEs filled the tunnel (Figures 6 and 7). The spatial density maps reveal a high similarity for the trajectories of DOPCs regardless the type of the tunnel-filling particle. The observed movement could be caused by the residence of neutral lipids inside the hydrophobic tunnel of CETP, since in order for CEs to properly accommodate the cavity, a conformational rearrangement of DOPCs would be required. Nonetheless, the wobbling of phospholipids with CETP-bound CE molecules indicates the structure of CETP to be rather unstable during the transportation of neutral lipids. This could highlight the importance of helix X needed to prevent the structure of the protein from collapsing, as was suggested also previously [4].

### The dynamical and structural analysis of CETP reveals conformational changes in helix X

The RMS fluctuations of CETP backbone were analyzed in order to find the regions that fluctuated the most during the simulations. This method of analysis can give valuable information regarding the functioning of a protein by highlighting the regions of protein backbone with low and high mobility.

**Figure 8:**
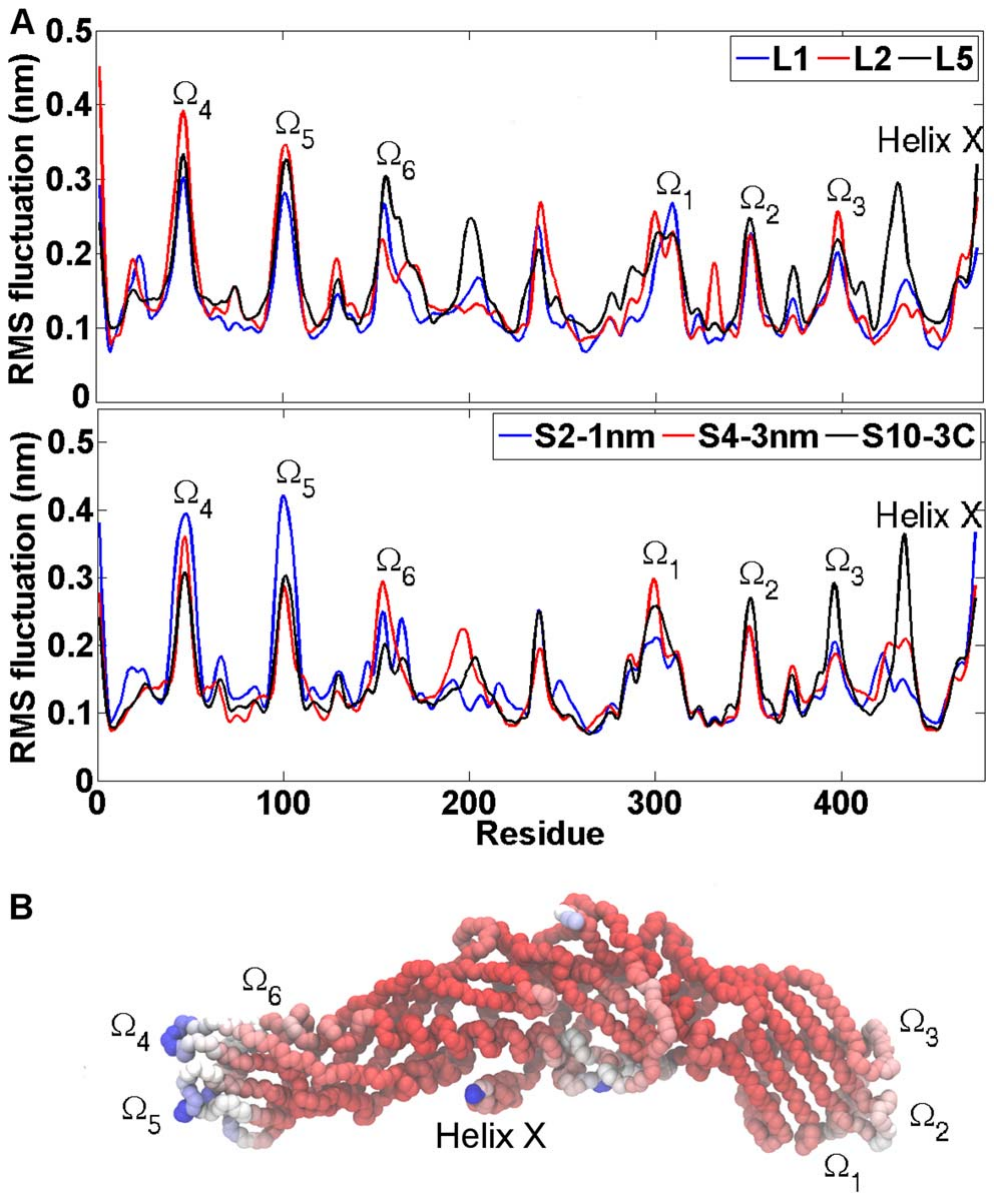
Dynamical properties of CETP. A) Residual RMS fluctuations for short 20 ns and long 200 ns simulations. The peaks indicate the regions of CETP that fluctuate the most over the course of simulations. These regions are found in loops marked with omegas as well as in the residues corresponding to helix X. B) Residual B-factors mapped to the backbone structure of CETP. Red color indicates the most rigid regions in the structure, whereas white and blue indicate the most flexible structural regions in the order of growing flexibility. Loop regions are marked with omegas and the region corresponding to helix X is labeled.

First, for comparison, CETP has previously been reported to have mobile structures with elevated B-factors near tunnel openings including the hinge region of helix X (Gly458-Pro460), and in the N- and C-terminal ends including the loop regions represented with omegas one and two [2], [4]. As expected, these regions showed high mobility also in our simulations (Figure 8), with the conformational fluctuation of helix X peaking near the residue 462. In addition, four other regions in the backbone of the protein were found to fluctuate highly during each simulation. These regions were Ω_3_ (residues 380–400), Ω_4_ (residues 40–50), Ω_5_ (residues 90–110) and Ω_6_ (residues 150–170) which were also earlier shown to have high mobility [4]. All these regions are found in the loops and therefore the high fluctuations can be expected. The results imply that the structure of CETP is elastic, facilitating the binding to lipoprotein surfaces with varying curvatures.

In addition, the observed flexibility of the hinge region of helix X (Glu 461-Ser472) suggests that helix X could play a crucial role in assisting the lipid exchange process. During the lipid exchange process helix X may partly move into the N-terminal CE-binding pocket of CETP to facilitate the export of CE out from CETP [4]. Another possibility is that helix X moves aside from the N-terminal tunnel opening, thus generating a wider pathway that facilitates the diffusion of CE out from CETP [22]. The above described free-energy calculations point to this direction.

**Figure 9:**
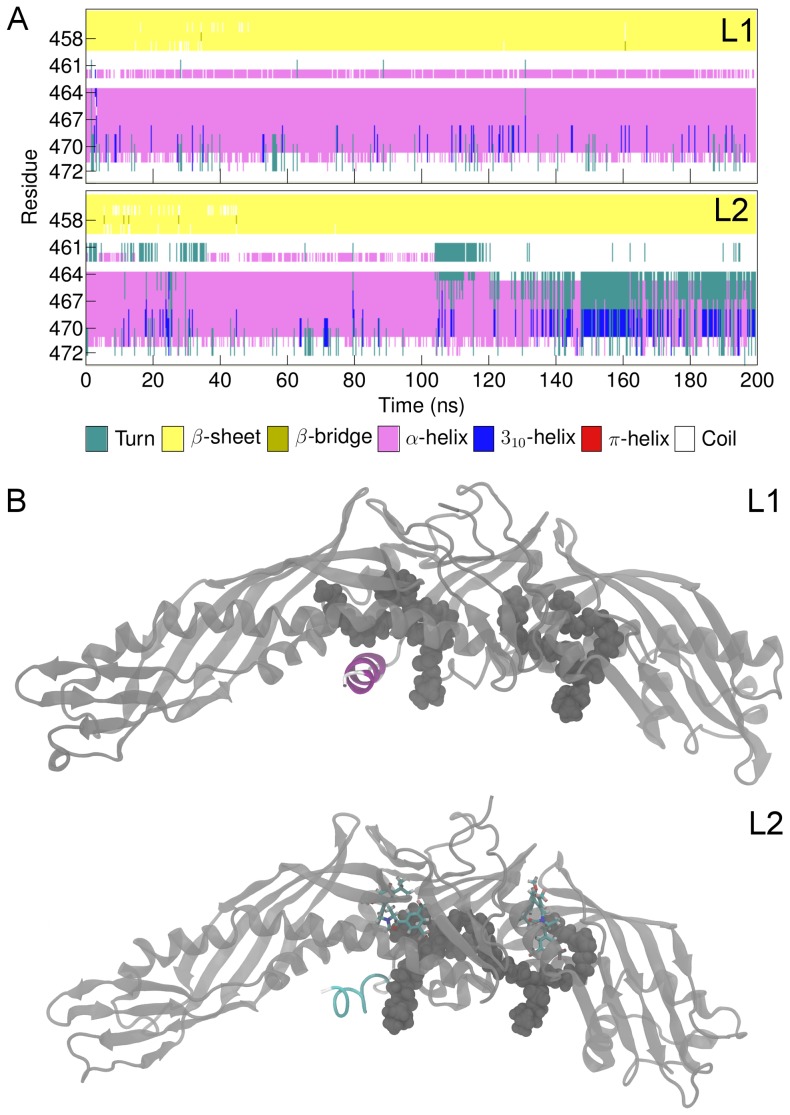
Conformational fluctuations of helix X. A) Secondary structure of helix X (residues 461–472) during simulations L1 and L2. B) Simulation snapshots from simulations L1 and L2. Helix X is colored to highlight the changes in the secondary structure. In the absence of anacetrapib (L1), helix X maintains the α-helical structure, whereas in the presence of anacetrapib (L2) it alternates between turn (unfolding of the helix) and 3_10_-helix (extension of the helix). Turn-like conformation is presented in the figure.

Interestingly, it became apparent based on DSSP calculations that the secondary structure of the helix encountered notable fluctuations between turn (unfolding of the helix) and 3_10_-helix (extension of the helix) when the drug molecule interacted with the concave surface of CETP (S1-helix, S2-1nm, S3-2nm, S7-1N, S8-2N, S9-1C), and when either CEs (L3, L5) or anacetrapibs (L2, L4) were present in the hydrophobic tunnel (Figure 9, Table 4). For comparison, helix X maintained its α-helical form during the simulation L1 where the hydrophobic tunnel was empty (Table 4). The visual inspection of the simulation trajectories revealed that in L1, the N-terminal DOPC maintained 1 nm distance from helix X over the course of simulation, while in L2 these two structures oriented themselves close to each other at the time when helix X experienced conformational fluctuations. The results imply that anacetrapib induces conformational alterations to the helix, and hence affects its stability, by interacting with the N-terminal DOPC. This, in turn, could indicate drastic effects on the lipid transfer functions of CETP.

**Table 4:** Percentage values of the different secondary structure elements of helix X in shorter and longer simulations.

Simulation	Coil	Bend	Turn	α-helix	3-Helix
S1-helix	0.33	0.03	0.13	0.50	0.01
S2-1nm	0.31	0.03	0.14	0.47	0.04
S3-2nm	0.32	0.02	0.14	0.49	0.04
S4-3nm	0.32	0.02	0.11	0.55	0.0
S5-4nm	0.35	0.09	0.14	0.40	0.01
S6-convex	0.33	0.04	0.14	0.47	0.01
S7-1N	0.34	0.02	0.13	0.50	0.02
S8-2N	0.33	0.04	0.15	0.47	0.01
S9-1C	0.33	0.04	0.18	0.12	0.32
S10-3C	0.31	0.07	0.13	0.47	0.02
L1	0.33	0.04	0.12	0.48	0.02
L2	0.35	0.12	0.17	0.31	0.04
L3	0.37	0.03	0.14	0.40	0.06
L4	0.34	0.18	0.18	0.24	0.05
L5	0.34	0.03	0.13	0.47	0.03

## Discussion

On the basis of earlier clinical trials, both anacetrapib and the flawed torcetrapib were shown to increase the binding affinity of CETP towards lipoproteins, especially towards HDL [1], [15], [19]. They induced a tight reversible binding on the lipoprotein surface stabilizing the HDL-CETP complex, and hereby preventing the capability of CETP to transport neutral lipids between different lipoprotein fractions. Despite the appealing start achieved in the anacetrapib-based CETP inhibition, the actual inhibitory mechanism of the drug remained unknown. In the present study, our objective was to reveal the mechanism of action behind anacetrapib, shed light on its ability to inhibit CETP-mediated lipid transfer, and to unravel the dynamics of related processes.

The results showed an evident affinity of anacetrapib towards the concave surface of CETP, especially towards the N-terminal tunnel opening where also helix X resides, and highlighted the importance of electrostatic and van der Waals interactions during the process once the drug was able to migrate to a close enough distance from the tunnel opening. However, the distance between the particles should be taken into consideration in the complex formation, since with too large distances (above about 3 nm) the movement of the drug was noticed to be dominated by thermal motion, eventually resulting in disordered motion. Hence, the question is how the affinity between the particles could be ensured in order to secure the interactions and speed up the complex formation process, as otherwise anacetrapib may experience random motion and may not be suitable for CETP inhibition purposes. For comparison, the formation of CETP-lipoprotein complex has been reported to be modulated by pH, surface pressure, and the introduction of positive divalent ions, such as Ca^2+^ and Mn^2+^, into the solution [23], [24]. In this spirit it is justified to assume that at least ion mediated interactions could foster the complex formation process, as electrostatics in terms of charged centers is an integral factor in both molecules but in the present case that we simulated in the absence of salt the screening effects were quite strong, resulting in predominance of thermal motion at long distances.

In addition to the observed affinity, anacetrapib was noticed to align itself to a tighter conformation while moving near the N-terminal opening. This finding together with the evidence of stronger interactions prevailing between the particles when the drug was transferred inside the hydrophobic cavity, indicate the primary binding site of anacetrapib to reside in the tunnel, particularly near the residues surrounding the N-terminal opening, including helix X. Hence, we propose CETP inhibition to be realized when the drug is transferred into the lipid binding pocket.

The regulatory role of helix X has been identified to play an important role in the lipid exchange process, since it has been suggested to act as a lid that conducts the exchange by alternating its open and closed states [4]. The structure of helix X has been proposed to undergo conformational changes during lipoprotein binding by moving aside from the N-terminal tunnel opening through an oblique penetration into the monolayer [22], or by rearranging and becoming buried inside the hydrophobic pocket [4]. Thus helix X is proposed to be locked in the “open” state for the time of lipid exchange. Considering the detachment as well as the transportation of lipids, helix X is needed to shield the corresponding tunnel opening to make CETP more compatible with the aqueous environment [2], hence the nomination “closed” state. The inhibitory mechanism of anacetrapib has been speculated to be in connection specifically with this regulatory property of helix X. One proposition suggests that CETP-bound anacetrapib alters the conformation of helix X to favor the open state, thus stabilizing the HDL-CETP –complex [4]. The above described findings disclose the flexible nature of helix X (the hinge region) that is essential in assisting the exchange of lipids. Our results are in agreement with these observations, as in the present simulations the hinge region of helix X was noticed to have elevated B-factors and, additionally, the secondary structure of the helix was shown to experience fluctuations between turn and 3_10_-helix while anacetrapib moved near the residues that surround the N-terminal tunnel opening. The results provide compelling evidence about the ability of anacetrapib to induce conformational changes to helix X in order to achieve the needed flexibility. How helix X behaves in the presence of the entire HDL-CETP-anacetrapib complex is a question left for additional simulations to be resolved.

Another crucial component in the process of neutral lipid exchange are the phospholipids due to their central role both in the binding and detachment of CETP from lipoprotein surfaces. During binding, phospholipids have been proposed to merge into the monolayer followed by a migration away from the tunnel openings to their edges [2], [4]. The molecular simulation data is strongly in favor of this view [4]. This process induces the formation of a hydrophobic pathway under the concave surface of the protein, which permits the access of lipids into the tunnel. Considering the detachment, the tunnel openings will need to be refilled with phospholipids before the dissociation since otherwise the protein would not be able to return to aqueous environment, or at least it would be much more unfavorable [2]. Hence, changes in the structure of phospholipids could possibly hinder their binding to CETP and the dissociation of CETP from lipoprotein surfaces, further resulting in a weaker capability of the protein to transport neutral lipids. Our results pointed to this direction, since phospholipids were noticed to experience increased structural fluctuations, in addition to the declined electrostatic and van der Waals interactions with CETP when anacetrapib was transferred into the hydrophobic tunnel of the protein. The corresponding interactions were stronger between phospholipids and CE molecules, suggesting the capability of the drug to destabilize the binding of phospholipids to CETP. This view is in accordance with the crystal structure of CETP published in complex with torcetrapib [17]. The structure indicates that the binding of torcetrapib to CETP abolishes the binding of phospholipids to the N-terminal tunnel opening. It is possible that torcetrapib together with CE excludes enough volume inside the hydrophobic tunnel of CETP rendering the binding of a phospholipid to the corresponding tunnel opening impossible, as the hydrophobic acyl chains of the phospholipid can no longer be buried inside CETP.

The primary binding site of anacetrapib to reside inside the hydrophobic tunnel is further supported by the free energy profiles that reveal strong attachment between the drug molecule and CETP, especially when two CEs fill the length of the tunnel as shown also previously [17]. When attached as described, anacetrapib hinders the ability of CE to diffuse out from the structure of CETP, thus pointing towards the possible inhibitory mechanism of the drug. The presence of helix X has a strong influence during this process, as both anacetrapib and CE were shown to move into the water phase more easily when the helix was removed from the structure of CETP. The results highlight the crucial role of helix X in assisting the lipid exchange during which helix X could possibly move aside from the N-terminal tunnel opening thus generating a wider pathway for CEs to diffuse out from CETP. It is reasonable that the hinge region of helix X enables the movement of the helix aside from the corresponding tunnel opening.

In conclusion, our results show an evident affinity of anacetrapib towards the concave surface of CETP, especially towards the region of N-terminal tunnel opening. The primary binding site for the drug turns out to reside inside the hydrophobic tunnel, near the residues surrounding the N-terminal opening. When residing in this area, anacetrapib was shown to hinder the ability of CE to diffuse out from the structure of CETP. Additionally, the results point towards the encouraging capability of anacetrapib to influence the molecular interactions between phospholipids and helix X, both of which represent the structural regions of CETP important for lipid exchange between lipoproteins, thus giving support for the competency of pharmacological CETP inhibition.

The view presented in this article paves the way for extending the scope of computational studies to gain a deeper understanding concerning the pharmacological ways to inhibit CETP and to modulate HDL levels. In this regard, simulations concerning the interactions between HDL-CETP-inhibitor –complex are ongoing (work in progress). The novel understanding could be used in the development of new molecular agents in the fight against the progression of cardiovascular diseases.

## Materials and Methods

### System setup and simulation details

Here we consider systems where anacetrapib interacts with CETP, which is either empty or carries a number of lipids in its transfer pocket. First, anacetrapib (Figure 1B) is an orally active, potent, and selective agent identified by high-throughput screening to belong to the 1–3-oxazolidin-2-one series of CETP inhibitors developed by Merck [15]. The medicinal chemistry behind the discovery of anacetrapib is described in [25]. The coordinate file for CETP in the PDB format was acquired from the RCSB Protein Databank with an accession code 2OBD. In addition to the protein, the file provides information of the atomic positions of the lipids involved in CETP: there are two CEs located inside the hydrophobic tunnel of CETP, and two DOPCs that cover the two openings of the tunnel. A detailed explanation of the protein structure is given elsewhere [2].

For all molecules considered in this study (CETP, DOPC, CE, anacetrapib), we used the official distribution force field OPLS-AA [26]–[33]. In addition, an extension of OPLS was used for the long hydrocarbon tails of DOPC and CE [34]. Concerning the partial charges, for DOPC molecules they were derived in compliance with the OPLS methodology [35], while for anacetrapib they were fitted to the electrostatic potential by applying the RESP software [36]. Here, the Merz-Kollman molecular electrostatic potential (MEP) was computed for the optimized structure of anacetrapib [37]. The MEP calculations were performed by applying the Gaussian 09 package at the Hartree-Fock level by employing the 6-31G* basis set [38]. The charges were fitted automatically by the RESP and ESP charge derived (R.E.D.) software version III. The derived charges can be found from the topology file Dataset S1 (see SI). Water molecules were described with the TIP3P model since it is compatible with the parametrization of OPLS-AA [39].

Prior to molecular dynamics simulations, we used molecular docking calculations to determine the initial configurations for the simulated systems. The purpose of the calculations was not to identify the possible binding poses of anacetrapib, but rather to explore the most probable sites from the crystal structure of CETP where the drug would desire to attach in terms of the lowest binding energy. The constructed box covered the two tunnel openings and the hydrophobic tunnel of CETP. CETP-bound lipids were not present. A flexible anacetrapib molecule with 9 rotatable bonds was used in the calculations. In total, 1000 runs were carried out with default settings, namely, the maximum number of binding modes to generate/export was set to 9, and the maximum energy difference between the best ligand pose and other ligand poses was set to 12.6 kJ mol^−1^. Four conformations with the highest binding free energy for anacetrapib are shown in Figure 1C. For ligands colored with red, brown, cyan, and green, the respective binding free energies were found to be −47.7 kJ mol^−1^, −46.4 kJ mol^−1^, −48.5 kJ mol^−1^, and −46.9 kJ mol^−1^. More detailed technical information for the applied program, AutoDock Vina, can be found in [40].

Based on the docking data (see Results for details; Figure 1C), we constructed initial simulation systems as divided into three groups. The first group consisted of 10 systems with lipids removed from CETP, and anacetrapib placed outside the protein but in the vicinity of its lipid binding pocket, to characterize the self-assembly process as well as to elucidate the interactions between the drug and the concave surface of the protein. In the first simulation (S1-helix), anacetrapib was placed 1 nm away from helix X, whereas in the four following systems (S2-1nm, S3-2nm, S4-3nm, S5-4nm) the drug was placed 1, 2, 3, and 4 nm from the tunnel openings, respectively. The sixth simulation (S6-convex) included anacetrapib at a distance of 3 nm from the convex back of the protein. The remaining four simulations (S7-1N, S8-2N, S9-1C, S10-3C) involved the drug at 1 and 2 nm distances from the N-terminal end, as well as 1 and 3 nm distances from the C-terminal end of the protein, respectively. All of the simulations in the first group were simulated for 20 ns each, thus “S” here in the system name stands for “short”.

The second group consisted of five systems with anacetrapib placed inside the lipid binding pocket to study the conformational changes of CETP induced by the drug. In the first simulation system (L1), the two CEs and anacetrapib were removed from CETP, thus only the two DOPC molecules remained inside the protein (Figure 2A). In the second simulation (L2, Figure 2B), two anacetrapib molecules were placed inside the empty hydrophobic tunnel based on the binding sites of cyan and green ligands presented in Figure 1C. Compared with L2, the drug molecules were replaced by two CEs in the third simulation (L3), the one being placed in the N-terminal domain and the other in the C-terminal domain of the protein (Figure 2C). Both L2 and L3 included DOPCs to plug the tunnel openings. All the lipids were removed from CETP in the fourth simulation (L4, Figure 2D), with one anacetrapib located in the empty tunnel between the binding sites determined by the cyan and green ligands (Figure 1C). In the fifth simulation (L5), the N-terminal DOPC was removed from CETP, with two CEs and one anacetrapib filling the length of the hydrophobic tunnel (Figure 2E). CEs were placed as in L3, while the location of anacetrapib was determined by the binding site of the cyan ligand (Figure 1C). Each of these systems, “L” standing for “long” ones, was simulated for 200 ns.

The third group consisted of eight systems directed to umbrella sampling simulations. In the first four systems, one anacetrapib molecule was pulled out from CETP through the N-terminal tunnel opening. The interior lipid composition and the presence of helix X in the structure of CETP were varied between the systems. In the first two systems, helix X was removed and the tunnel was either empty (U1, Figure 2F) or contained two CEs (U2, Figure 2G). Helix X was returned to CETP in the following two simulations and the tunnel was both empty (U3, Figure 2H) and filled with two CEs (U4, Figure 2I). In all the remaining four systems, two CEs resided inside the hydrophobic tunnel, the N-terminal CE being the molecule pulled out from the structure of CETP with the varying presence of helix X and one anacetrapib. Both of these were absent in the fifth simulation (U5, Figure 2J), while in the sixth simulation (U6) anacetrapib was added inside the tunnel, near the N-terminal tunnel opening (Figure 2K). Helix X was returned to CETP in the last two simulations without (U7) and with (U8) one anacetrapib locating near the N-terminal tunnel opening (Figure 2L and 2M). The N-terminal DOPC molecule was removed in all eight systems, whereas the C-terminal DOPC plugged the corresponding tunnel opening. Each of these systems, “U” standing for umbrella sampling (see below), were first equilibrated for 100 ns, and then simulated for 60 ns.

The systems belonging to the first group were solvated with ∼100,000–160,000 water molecules with counter ions, while in the second and third groups about 100,000 and ∼30,000–60,000 water molecules, respectively, were used for solvation. Altogether, the systems included ∼125,000–500,000 atoms. The number of water molecules used in the simulations depends on the size of the simulation box: the greater the distance between CETP and anacetrapib, the greater the size of the simulation box and, hence, the number of water molecules. The size of the simulation box originates from the requirement of periodic boundary conditions. Before any simulation was started, energy minimization was performed for each system using the steepest descent method with 500 steps [41].

Prior to conducting umbrella sampling simulations, eight pulling simulations were performed in order to generate a series of configurations that served as the starting configurations for umbrella sampling. In the pulling simulations, either anacetrapib or the N-terminal CE, depending on the system, was pulled out from the structure of CETP through the N-terminal tunnel opening. Residues Cysh9, Arg10, Ile11, and Thr12 were used as the reference pull group due to their parallel location with the N-terminal tunnel opening. The force constant applied either to the center of mass of anacetrapib or to the last carbon atom in the acyl chain of CE was 2000 kJ mol^−1^ nm^−2^ with a pull rate of 0.003 nm ns^−1^. After pulling simulations, umbrella sampling was conducted with a total of 25 and 47 umbrella windows when the pulled molecule was anacetrapib and CE, respectively. The umbrella windows were selected at 0.1 nm intervals from the original location of the molecules inside the hydrophobic tunnel towards the water phase. Each window was simulated for 160 ns where the first 100 ns were used for equilibration. These parameters were chosen based on a systematic study for increasing equilibration and simulation times. Altogether, the umbrella sampling simulations required about 600 core-years of computing time. In these simulations, both molecules were restrained to the middle of every umbrella window by a harmonic potential with the same force constant as used in the pulling simulations. The restraints were applied only along the reaction coordinate defined by the vector connecting the reference pull group and the pulled molecule, and the molecules were free to move in the other directions. In order to keep the reaction coordinate along the vector and to prevent CETP from rotating and tilting, position restraints were applied to a carbon atom of Ser115 and to a carbon atom Gly413. These atoms reside in the N- and C-terminal ends of the protein, respectively.

The simulations were performed under NpT conditions (constant number of particles, pressure, and temperature) with the GROMACS software package using the version 4.5.4 for the first and second simulation groups and version 4.6.1 for the third simulation group [41]. The reference temperature for all simulated systems was 310 K, and each component of the systems was separately coupled to a temperature bath using the Nόse-Hoover coupling method with a time constant of 0.1 ps [42]. The Parrinello-Rahman barostat was applied to couple the pressure, with a coupling constant of 1 ps and a reference pressure of 1 bar [43]. A time step of 2 fs was used in all simulations, with the LINCS algorithm applied to constrain all the bonds in the system [44]. The van der Waals interactions were calculated up to a cutoff radius of 1 nm and the particle mesh Ewald technique was utilized for long-range Coulombic forces, with a real space cutoff of 1 nm [45], [46]. As mentioned above, the ten systems associated with the first simulation group (“S” standing for short) were simulated for 20 ns, the systems in the second group (“L” standing for long) for 200 ns, and the systems in the third group (“U” standing for umbrella sampling) for 160 ns where the first 100 ns were used for equilibration.

### Analysis methods

DSSP (define secondary structure of proteins) [47] was applied to determine the most likely secondary structure of CETP as a function of time. DSSP was calculated by applying the do_dssp tool of GROMACS.

The root mean square deviation (RMSD) was used to evaluate the deviation of the structure of the simulated system from the initial starting structure over the course of simulation. RMSD was calculated by applying the g_rms tool of GROMACS.

The radius of gyration was used to measure the size and compactness of a molecule. It is defined as the mean square distance of each particle in the structure with respect to its center of mass. The radius of gyration was calculated by applying the g_gyrate tool of GROMACS.

The root mean square fluctuation (RMSF) of atomic positions was used to discover and evaluate the most flexible regions of CETP. RMSFs were calculated by fitting the simulated structure to a reference structure followed by the calculation of the average distance deviation from the reference structure. Typically, the residues of a protein that fluctuate the most can be found in loop regions. For purposes of comparison with experimental data, the RMSFs can be converted into B-factor values. RMSF was calculated by applying the g_rmsf tool of GROMACS.

Interaction energies between different molecules were calculated by applying the g_energy tool of GROMACS. The tool calculates the contributions of the energies automatically from the simulation trajectory.

Weighted histogram analysis method (WHAM) is a standard technique to compute the potential of mean force (PMF) along the reaction coordinate for a molecule [48], [49]. It estimates the statistical uncertainty of the probability distribution obtained from the umbrella histograms and iteratively computes the PMF that corresponds to the smallest uncertainty in the form of global free energy of the molecule [49]. WHAM was calculated by applying the g_wham tool of GROMACS.

Statistical uncertainty of the PMF can be estimated by applying the bootstrap analysis. The idea is to generate new hypothetical observations, that is, a bootstrapped trajectory for each umbrella histogram, thus yielding a new set of histograms for the corresponding umbrella window that are subsequently applied in WHAM to calculate a bootstrapped PMF. The process is repeated for each bootstrapped trajectory when a large number of bootstrapped PMFs can be obtained. The applied bootstrapped sample size was 200.

Hydrogen bond formation has a remarkable role, e.g., in the stabilization of the secondary structure of a molecule. In this study the formation of hydrogen bonds is analyzed between CETP and anacetrapib in order to see where and how tightly anacetrapib binds. The formation of hydrogen bonds is analyzed between all possible donors D and acceptors A. OH and NH groups are regarded as donors while O is always an acceptor. The determination for the existence of a hydrogen bond is done by using a geometrical criterion (based on the distance between donor-acceptor *r*_DA_) and a criterion for the angle *α* (between acceptor-donor-hydrogen triplet *α*_ADH_),
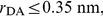
(1)

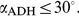
(2)Hydrogen bonds were calculated by applying the g_hbond tool of GROMACS.

## Supporting Information

Figure S1
**Movement of anacetrapib around CETP.** Spatial density maps for anacetrapibs involved in the MD simulations performed for 20 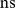. The map is colored with gray revealing the movement of the drug outside the lipid binding pocket of CETP.(DOC)

Dataset S1 **Derived charges for anacetrapib, based on its topology file.**
(DOC)
