# Supplementary material for: How Anacetrapib Inhibits the Activity of the Cholesteryl Ester Transfer Protein? Perspective through Atomistic Simulations
Source: PLoS Comput Biol. 2014 Nov 20;10(11):e1003987. doi: 10.1371/journal.pcbi.1003987 (PMC4238956; doi:10.1371/journal.pcbi.1003987)
Supplement: Figure S1 — Movement of anacetrapib around CETP. Spatial density maps for anacetrapibs involved in the MD simulations performed for 20 . The map is colored with gray revealing the movement of the drug outside the lipid binding pocket of CETP. (DOC) [file pcbi.1003987.s001.doc]

**How Anacetrapib Inhibits the Activity of the Cholesteryl Ester Transfer Protein? Perspective through Atomistic Simulations**

Tarja Äijänen, Artturi Koivuniemi, Matti Javanainen, Sami Rissanen, Tomasz Rog, Ilpo Vattulainen

**Supporting Information**

**
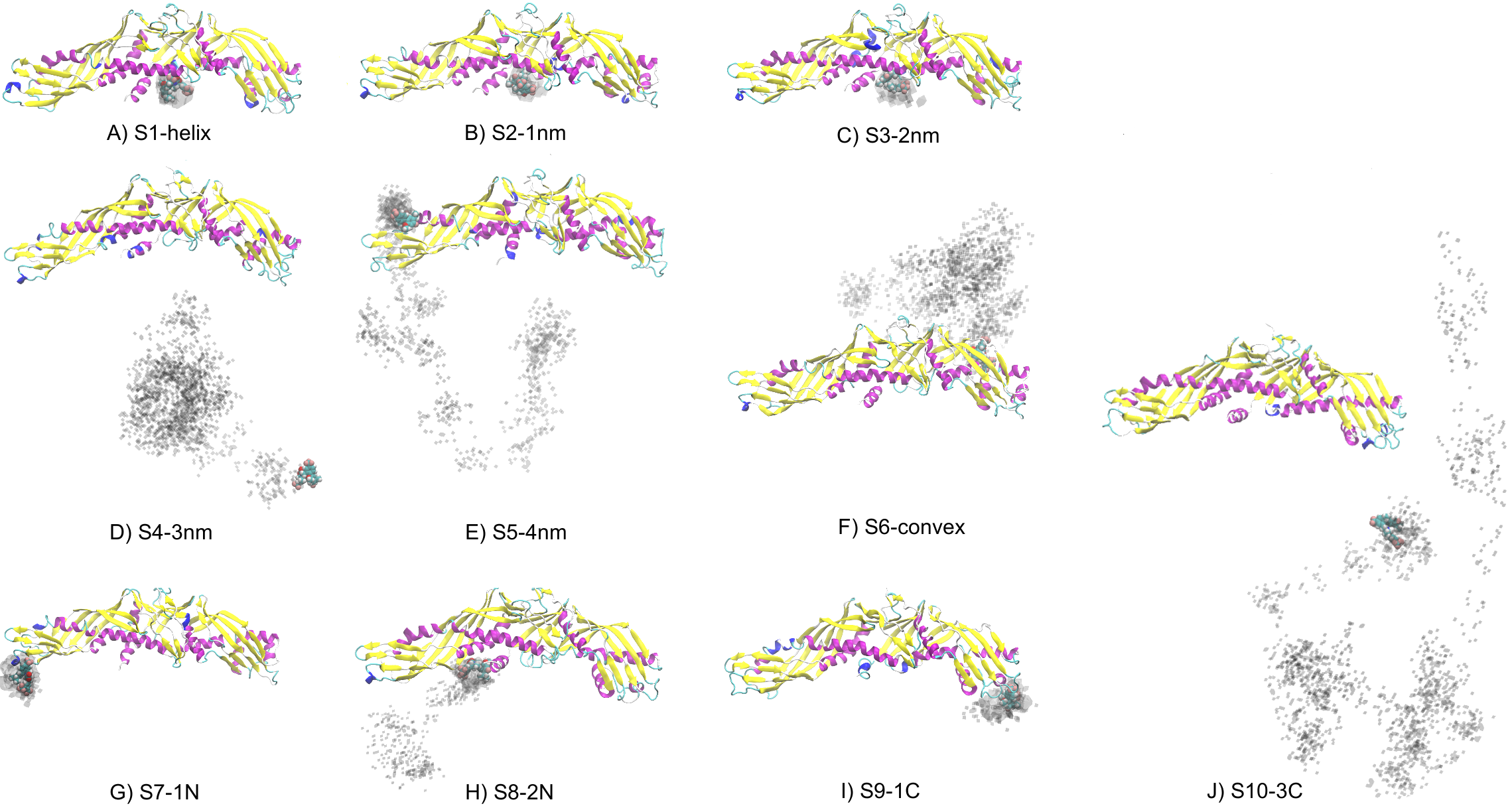
**

**Figure S1. Movement of anacetrapib around CETP.** Spatial density maps for anacetrapibs involved in the MD simulations performed for 20$\text{ns}$. The map is colored with gray revealing the movement of the drug outside the lipid binding pocket of CETP.
