# Supplementary material for: How Anacetrapib Inhibits the Activity of the Cholesteryl Ester Transfer Protein? Perspective through Atomistic Simulations
Source: PLoS Comput Biol. 2014 Nov 20;10(11):e1003987. doi: 10.1371/journal.pcbi.1003987 (PMC4238956; doi:10.1371/journal.pcbi.1003987)
Supplement: Dataset S1 — Derived charges for anacetrapib, based on its topology file. (DOC) [file pcbi.1003987.s002.doc]

**How Anacetrapib Inhibits the Activity of the Cholesteryl Ester Transfer Protein? Perspective through Atomistic Simulations**

Tarja Äijänen, Artturi Koivuniemi, Matti Javanainen, Sami Rissanen, Tomasz Rog, Ilpo Vattulainen

**Supporting Information**

**Dataset S1. Force field of anacetrapib used in the molecular dynamics simulations.**

;

;

; H

; |

; H - C - H

; |

; H H O H

; \ / \ /

; F C - C C - C

; \ / \ / \

; F - C - C C - C C - F

; / \ / \ /

; F H - C - C H C - C H H

; \ / / \ / /

; O C H C - C - H

; \\ / \ | \

; C - N H H H-C-H H

; / \ / |

; O C - C - H H

; \ / \ \

; H - C H H

; |

; H C H

; \ / \ /

; F C C F

; \ | | /

; F - C - C C - C - F

; / \ / \

; F C F

; |

; H

;

;

; 3

; |

; 2 - 1 - 4

; |

; 29 27 5 8

; \ / \ /

; 34 28 - 26 6 - 7

; \ / \ / \

; 33 - 31- 30 25 - 24 9 - 10

; / \ / \ /

; 32 36-35 - 37 39 22 - 11 13 15

; \ / / \ / /

; 49 38 23 12 - 14 - 16

; \\ / \ | \

; 48 -41 40 45 21-18-19 17

; / \ / |

; 50 42 - 44-46 20

; \ / \ \

; 52 - 51 43 47

; |

; 55 53 57

; \ / \ /

; 69 54 56 60

; \ | | /

; 68 - 66-65 58 - 59 - 61

; / \ / \

; 67 63 62

; |

; 64

;

[ moleculetype ]

;

ANACE 3

;

[ atoms ]

; nbr type resnb residu atom chgrpnb charge mass

1 opls_135 1 AN1 C1 1 -0.044242 12.01100

2 opls_140 1 AN1 H1 1 0.081955 1.00800

3 opls_140 1 AN1 H2 1 0.081955 1.00800

4 opls_140 1 AN1 H3 1 0.081955 1.00800

5 opls_179 1 AN1 O1 1 -0.272411 15.99940

6 opls_199 1 AN1 C2 1 0.170642 12.01100

7 opls_145 1 AN1 C3 2 -0.223759 12.01100

8 opls_146 1 AN1 H4 2 0.156204 1.00800

9 opls_145 1 AN1 C4 3 0.102343 12.0110

10 opls_719 1 AN1 F1 3 -0.142210 18.99840

11 opls_145 1 AN1 C5 4 0.002176 12.01100

12 opls_137 1 AN1 C6 5 0.182819 12.01100

13 opls_140 1 AN1 H5 5 0.018731 1.00800

14 opls_135 1 AN1 C7 6 -0.252364 12.01100

15 opls_140 1 AN1 H6 6 0.065001 1.00800

16 opls_140 1 AN1 H7 6 0.065001 1.00800

17 opls_140 1 AN1 H8 6 0.065001 1.00800

18 opls_135 1 AN1 C8 7 -0.252364 12.01100

19 opls_140 1 AN1 H9 7 0.065001 1.00800

20 opls_140 1 AN1 H10 7 0.065001 1.00800

21 opls_140 1 AN1 H11 7 0.065001 1.00800

22 opls_145 1 AN1 C9 8 -0.270439 12.01100

23 opls_146 1 AN1 H12 8 0.214039 1.00800

24 opls_145 1 AN1 C10 9 0.018727 12.01100

25 opls_145 1 AN1 C11 10 0.064240 12.01100

26 opls_145 1 AN1 C12 11 -0.254071 12.01100

27 opls_146 1 AN1 H13 11 0.183384 1.00800

28 opls_145 1 AN1 C13 12 -0.101875 12.01100

29 opls_146 1 AN1 H14 12 0.151268 1.00800

30 opls_724 1 AN1 C14 13 0.001312 12.01100

31 opls_725 1 AN1 C15 13 0.485410 12.01100

32 opls_726 1 AN1 F2 13 -0.184372 18.99840

33 opls_726 1 AN1 F3 13 -0.184372 18.99840

34 opls_726 1 AN1 F4 13 -0.184372 18.99840

35 opls_145 1 AN1 C16 14 -0.208975 12.01100

36 opls_146 1 AN1 H15 14 0.123250 1.00800

37 opls_145 1 AN1 C17 15 0.015295 12.01100

38 opls_137 1 AN1 C18 16 0.004264 12.01100

39 opls_140 1 AN1 H16 16 0.064458 1.00800

40 opls_140 1 AN1 H17 16 0.064458 1.00800

41 opls_239 1 AN1 N1 16 -0.066751 14.00670

42 opls_137 1 AN1 C19 16 -0.004717 12.01100

43 opls_140 1 AN1 H18 16 0.066940 1.00800

44 opls_135 1 AN1 C20 17 -0.357666 12.01100

45 opls_140 1 AN1 H19 17 0.113825 1.00800

46 opls_140 1 AN1 H20 17 0.113825 1.00800

47 opls_140 1 AN1 H21 17 0.113825 1.00800

48 opls_235 1 AN1 C21 18 0.653567 12.01100

49 opls_236 1 AN1 O2 18 -0.554449 15.99940

50 opls_467 1 AN1 O3 18 -0.377472 15.99940

51 opls_137 1 AN1 C22 18 0.164698 12.01100

52 opls_140 1 AN1 H22 18 0.071804 1.00800

53 opls_145 1 AN1 C23 18 0.004923 12.01100

54 opls_145 1 AN1 C24 19 -0.114794 12.01100

55 opls_146 1 AN1 H23 19 0.162539 1.00800

56 opls_145 1 AN1 C25 20 -0.114794 12.01100

57 opls_146 1 AN1 H24 20 0.162539 1.00800

58 opls_724 1 AN1 C26 21 -0.052680 12.01100

59 opls_725 1 AN1 C27 21 0.578297 12.01100

60 opls_726 1 AN1 F5 21 -0.198211 18.99840

61 opls_726 1 AN1 F6 21 -0.198211 18.99840

62 opls_726 1 AN1 F7 21 -0.198211 18.99840

63 opls_145 1 AN1 C28 22 -0.170990 12.01100

64 opls_146 1 AN1 H25 22 0.188115 1.00800

65 opls_724 1 AN1 C29 23 -0.052680 12.01100

66 opls_725 1 AN1 C30 23 0.578297 12.01100

67 opls_726 1 AN1 F8 23 -0.198211 18.99840

68 opls_726 1 AN1 F9 23 -0.198211 18.99840

69 opls_726 1 AN1 F10 23 -0.198211 18.99840

;

[ bonds ]

; ai aj funct

1 2 1

1 3 1

1 4 1

1 5 1

5 6 1

6 7 1

6 24 1

7 8 1

7 9 1

9 10 1

9 11 1

11 12 1

11 22 1

12 13 1

12 14 1

12 18 1

14 15 1

14 16 1

14 17 1

18 19 1

18 20 1

18 21 1

22 23 1

22 24 1

24 25 1

25 26 1

25 37 1

26 27 1

26 28 1

28 29 1

28 30 1

30 31 1

30 35 1

31 32 1

31 33 1

31 34 1

35 36 1

35 37 1

37 38 1

38 39 1

38 40 1

38 41 1

41 42 1

41 48 1

42 43 1

42 44 1

42 51 1

44 45 1

44 46 1

44 47 1

48 49 1

48 50 1

50 51 1

51 52 1

51 53 1

53 54 1

53 56 1

54 55 1

54 65 1

56 57 1

56 58 1

58 59 1

58 63 1

59 60 1

59 61 1

59 62 1

63 64 1

63 65 1

65 66 1

66 67 1

66 68 1

66 69 1

[ angles ]

; ai aj ak funct

2 1 3 1

2 1 4 1

2 1 5 1

3 1 4 1

3 1 5 1

4 1 5 1

1 5 6 1

5 6 7 1

5 6 24 1

7 6 24 1

6 7 8 1

6 7 9 1

8 7 9 1

7 9 10 1

7 9 11 1

10 9 11 1

9 11 12 1

9 11 22 1

12 11 22 1

11 12 13 1

11 12 14 1

11 12 18 1

13 12 14 1

13 12 18 1

14 12 18 1

12 14 15 1

12 14 16 1

12 14 17 1

15 14 16 1

15 14 17 1

16 14 17 1

12 18 19 1

12 18 20 1

12 18 21 1

19 18 20 1

19 18 21 1

20 18 21 1

11 22 23 1

11 22 24 1

23 22 24 1

6 24 22 1

6 24 25 1

22 24 25 1

24 25 26 1

24 25 37 1

26 25 37 1

25 26 27 1

25 26 28 1

27 26 28 1

26 28 29 1

26 28 30 1

29 28 30 1

28 30 31 1

28 30 35 1

31 30 35 1

30 31 32 1

30 31 33 1

30 31 34 1

32 31 33 1

32 31 34 1

33 31 34 1

30 35 36 1

30 35 37 1

36 35 37 1

25 37 35 1

35 37 38 1

35 37 38 1

37 38 39 1

37 38 40 1

37 38 41 1

39 38 40 1

39 38 41 1

40 38 41 1

38 41 42 1

38 41 48 1

42 41 48 1

41 42 43 1

41 42 44 1

41 42 51 1

43 42 44 1

43 42 51 1

44 42 51 1

42 44 45 1

42 44 46 1

42 44 47 1

45 44 46 1

45 44 47 1

46 44 47 1

41 48 49 1

41 48 50 1

49 48 50 1

48 50 51 1

42 51 50 1

42 51 52 1

42 51 53 1

50 51 52 1

50 51 53 1

52 51 53 1

51 53 54 1

51 53 56 1

54 53 56 1

53 54 55 1

53 54 65 1

55 54 65 1

53 56 57 1

53 56 58 1

57 56 58 1

56 58 59 1

56 58 63 1

59 58 63 1

58 59 60 1

58 59 61 1

58 59 62 1

60 59 61 1

60 59 62 1

61 59 62 1

58 63 64 1

58 63 65 1

64 63 65 1

54 65 63 1

54 65 66 1

63 65 66 1

65 66 67 1

65 66 68 1

65 66 69 1

67 66 68 1

67 66 69 1

68 66 69 1

;

[ dihedrals ]

; a1 a2 a3 a4 funct

2 1 5 6 3

3 1 5 6 3

4 1 5 6 3

1 5 6 7 3

1 5 6 24 3

5 6 7 8 3

5 6 7 9 3

24 6 7 8 3

24 6 7 9 3

5 6 24 22 3

5 6 24 25 3

7 6 24 22 3

7 6 24 25 3

6 7 9 10 3

6 7 9 11 3

8 7 9 10 3

8 7 9 11 3

7 9 11 12 3

7 9 11 22 3

10 9 11 12 3

10 9 11 22 3

9 11 12 13 3

9 11 12 14 3

9 11 12 18 3

22 11 12 13 3

22 11 12 14 3

22 11 12 18 3

9 11 22 23 3

9 11 22 24 3

12 11 22 23 3

12 11 22 24 3

11 12 14 15 3

11 12 14 16 3

11 12 14 17 3

13 12 14 15 3

13 12 14 16 3

13 12 14 17 3

18 12 14 15 3

18 12 14 16 3

18 12 14 17 3

11 12 18 19 3

11 12 18 20 3

11 12 18 21 3

13 12 18 19 3

13 12 18 20 3

13 12 18 21 3

14 12 18 19 3

14 12 18 20 3

14 12 18 21 3

11 22 24 6 3

11 22 24 25 3

23 22 24 6 3

23 22 24 25 3

6 24 25 26 3

6 24 25 37 3

22 24 25 26 3

22 24 25 37 3

24 25 26 27 3

24 25 26 28 3

37 25 26 27 3

37 25 26 28 3

24 25 37 35 3

24 25 37 38 3

26 25 37 35 3

26 25 37 38 3

25 26 28 29 3

25 26 28 30 3

27 26 28 29 3

27 26 28 30 3

26 28 30 31 3

26 28 30 35 3

29 28 30 31 3

29 28 30 35 3

28 30 31 32 3

28 30 31 33 3

28 30 31 34 3

35 30 31 32 3

35 30 31 33 3

35 30 31 34 3

28 30 35 36 3

28 30 35 37 3

31 30 35 36 3

31 30 35 37 3

30 35 37 25 3

30 35 37 38 3

36 35 37 25 3

36 35 37 38 3

25 37 38 39 3

25 37 38 40 3

25 37 38 41 3

35 37 38 39 3

35 37 38 40 3

35 37 38 41 3

37 38 41 42 3

37 38 41 48 3

39 38 41 42 3

39 38 41 48 3

40 38 41 42 3

40 38 41 48 3

38 41 42 43 3

38 41 42 44 3

38 41 42 51 3

48 41 42 43 3

48 41 42 44 3

48 41 42 51 3

38 41 48 49 3

38 41 48 50 3

42 41 48 49 3

42 41 48 50 3

41 42 44 45 3

41 42 44 46 3

41 42 44 47 3

43 42 44 45 3

43 42 44 46 3

43 42 44 47 3

51 42 44 45 3

51 42 44 46 3

51 42 44 47 3

41 42 51 50 3

41 42 51 52 3

41 42 51 53 3

43 42 51 50 3

43 42 51 52 3

43 42 51 53 3

44 42 51 50 3

44 42 51 52 3

44 42 51 53 3

41 48 50 51 3

49 48 50 51 3

48 50 51 42 3

48 50 51 52 3

48 50 51 53 3

42 51 53 54 3

42 51 53 56 3

50 51 53 54 3

50 51 53 56 3

52 51 53 54 3

52 51 53 56 3

51 53 56 57 3

51 53 56 58 3

54 53 56 57 3

54 53 56 58 3

51 53 54 55 3

51 53 54 65 3

56 53 54 55 3

56 53 54 65 3

53 54 65 63 3

53 54 65 66 3

55 54 65 63 3

55 54 65 66 3

53 56 58 59 3

53 56 58 63 3

57 56 58 59 3

57 56 58 63 3

56 58 59 60 3

56 58 59 61 3

56 58 59 62 3

63 58 59 60 3

63 58 59 61 3

63 58 59 62 3

56 58 63 64 3

56 58 63 65 3

59 58 63 64 3

59 58 63 65 3

58 63 65 54 3

58 63 65 66 3

64 63 65 54 3

64 63 65 66 3

54 65 66 67 3

54 65 66 68 3

54 65 66 69 3

63 65 66 67 3

63 65 66 68 3

63 65 66 69 3

;

[ pairs ]

; a1 a4 funct

2 6 1

3 6 1

4 6 1

1 7 1

1 24 1

5 8 1

5 9 1

24 8 1

24 9 1

5 22 1

5 25 1

7 22 1

7 25 1

6 10 1

6 11 1

8 10 1

8 11 1

7 12 1

7 22 1

10 12 1

10 22 1

9 13 1

9 14 1

9 18 1

22 13 1

22 14 1

22 18 1

9 23 1

9 24 1

12 23 1

12 24 1

11 15 1

11 16 1

11 17 1

13 15 1

13 16 1

13 17 1

18 15 1

18 16 1

18 17 1

11 19 1

11 20 1

11 21 1

13 19 1

13 20 1

13 21 1

14 19 1

14 20 1

14 21 1

11 6 1

11 25 1

23 6 1

23 25 1

6 26 1

6 37 1

22 26 1

22 37 1

24 27 1

24 28 1

37 27 1

37 28 1

24 35 1

24 38 1

26 35 1

26 38 1

25 29 1

25 30 1

27 29 1

27 30 1

26 31 1

26 35 1

29 31 1

29 35 1

28 32 1

28 33 1

28 34 1

35 32 1

35 33 1

35 34 1

28 36 1

28 37 1

31 36 1

31 37 1

30 25 1

30 38 1

36 25 1

36 38 1

25 39 1

25 40 1

25 41 1

35 39 1

35 40 1

35 41 1

37 42 1

37 48 1

39 42 1

39 48 1

40 42 1

40 48 1

38 43 1

38 44 1

38 51 1

48 43 1

48 44 1

48 51 1

38 49 1

38 50 1

42 49 1

42 50 1

41 45 1

41 46 1

41 47 1

43 45 1

43 46 1

43 47 1

51 45 1

51 46 1

51 47 1

41 50 1

41 52 1

41 53 1

43 50 1

43 52 1

43 53 1

44 50 1

44 52 1

44 53 1

41 51 1

49 51 1

48 42 1

48 52 1

48 53 1

42 54 1

42 56 1

50 54 1

50 56 1

52 54 1

52 56 1

51 57 1

51 58 1

54 57 1

54 58 1

51 55 1

51 65 1

56 55 1

56 65 1

53 63 1

53 66 1

55 63 1

55 66 1

53 59 1

53 63 1

57 59 1

57 63 1

56 60 1

56 61 1

56 62 1

63 60 1

63 61 1

63 62 1

56 64 1

56 65 1

59 64 1

59 65 1

58 54 1

58 66 1

64 54 1

64 66 1

54 67 1

54 68 1

54 69 1

63 67 1

63 68 1

63 69 1
